# Evaluating traditional Chinese medicine modulation of the gut microbiota–bile acid axis in metabolic and inflammatory diseases: mechanistic evidence and translational priorities

**DOI:** 10.3389/fmicb.2026.1977691

**Published:** 2026-10-13

**Authors:** Yanyan Yu, Jian Wang, Qingbao Yuan, Linghui Yu, Chunling Zhang

**Affiliations:** 1 Qingdao Central Hospital, University of Health and Rehabilitation Sciences, Qingdao, China; 2 Qingdao Special Service Sanatorium of PLA Navy, Qingdao, Shandong, China; 3 Shandong University of Traditional Chinese Medicine Affiliated Hospital, Jinan, Shandong, China

**Keywords:** bile acid metabolism, gut microbiota-bile acids axis, inflammatory bowel disease, metabolic dysfunction-associated steatotic liver disease (MALSD), obesity, traditional Chinese medicine, translational pathway validation, type 2 diabetes mellitus

## Abstract

Metabolic dysfunction-associated steatotic liver disease (MASLD), obesity, type 2 diabetes mellitus (T2D), and inflammatory bowel disease (IBD) are clinically distinct disorders that share perturbations in intestinal ecology, bile-acid composition, epithelial barrier function, and immune-metabolic regulation. Traditional Chinese medicine (TCM) may modify these interconnected processes, but the translational significance of reported effects remains uncertain. Changes in microbial composition are often interpreted as mechanisms without evidence of altered microbial function, bile-acid profiles or concentrations, receptor engagement, or clinical benefit. Here, we present a structured narrative review that uses the gut microbiota-bile acid axis to evaluate TCM in metabolic and inflammatory disease. We describe how microbial bile salt hydrolases, the bile acid-inducible (*bai*) operon, hydroxysteroid dehydrogenases, and amino-acid conjugation diversify the bile-acid pool and influence farnesoid X receptor (FXR), Takeda G protein-coupled bile acid receptor 1 (GPBAR1, also known as TGR5), and immune pathways. We then assess evidence for defined TCM constituents and formulae in MASLD, obesity/T2D, and IBD. Across these settings, the most informative studies combine a standardized intervention with longitudinal metagenomic and targeted bile-acid profiling, host-signaling measurements, and an outcome that is attenuated by microbiota depletion, fecal microbiota transplantation, or functional microbial manipulation. Only one eligible human trial, of Qushi Huayu in adults enrolled under the historical NAFLD designation, reported both clinical and microbiota-related outcomes. Evidence for obesity/T2D and IBD was predominantly preclinical or observational, and the framework is therefore a translational assessment tool rather than a clinically validated model. We therefore propose an evidence hierarchy and a minimum translational package that integrates chemical quality control, dietary and co-medication assessment, strain- and gene-resolved microbiome analysis, targeted bile-acid metabolomics, and pathway-directed validation. TCM should be considered a potential precision adjunct to standard care only when reproducible microbial-metabolic phenotypes are linked to clinically meaningful benefit.

## 1 Introduction

MASLD, obesity, T2D, and IBD share altered bile-acid composition, barrier dysfunction, and immune-metabolic disturbance, but their relative contributions vary by disease, site, and stage (Mohanty et al., 2024). The gut microbiota-bile acid axis links diet, liver metabolism, microbes, and host signalling. Proposed mechanisms should define the relevant species, compartment, and host response. MASLD is the canonical term; NAFLD is retained only for studies using the historical designation and is not treated as interchangeable.

Most primary bile acids are conjugated in the liver, whereas intestinal microbes deconjugate, dehydroxylate, oxidize, and epimerize the bile-acid pool. After enterohepatic return, hepatocytes predominantly reconjugate bile acids with glycine or taurine; microbial amino-acid conjugation is a distinct luminal transformation. Candidate functions include BSHs, the *bai* operon, hydroxysteroid dehydrogenases, and microbial amino-acid conjugation. Diet, transport, inflammation, hepatic production, treatment, and surgery also shape the system, so taxa and fecal bile acids may reflect host selection or altered delivery, reabsorption, dilution, and transformation. Functional claims require more than relative abundance (Guzior and Quinn, 2021).

Traditional Chinese medicine (TCM) is rich in chemically diverse polysaccharides, flavonoids, alkaloids and saponins, which can be metabolized by the gut microbial community (Zhang H. Y. et al., 2021). Formulae may change microbial substrates and yield metabolites having different absorption or receptor activity, but may also have a direct action on host tissues. Botanical identity, extraction, dose, and batch consistency determine intestinal exposure and reproducibility; microbiota changes alone do not distinguish direct from microbiota-dependent pharmacology.

Parallel changes in a bacterial taxon, a bile acid and a disease biomarker may generate a mechanistic hypothesis, but they do not establish mediation. Interpretation therefore requires a characterized intervention, a defined microbial or metabolic intermediate, a host response, and a disease-relevant outcome measured in an appropriate temporal and biological context.

Context limits simple interpretation. In MASLD and obesity/T2D, diet, weight, incretins, and drugs can change microbial and bile-acid profiles independently of clinical improvement. In IBD, inflammation, ileal involvement, nutrition, medicines, and surgery alter bile-acid absorption and microbial ecology. Stool may not reflect ileal, mucosal, hepatic, or systemic exposure, and increased secondary bile acids or intestinal FXR activity is not uniformly beneficial (Nie et al., 2024).

Mechanistic analysis must evolve from named genera to measurable functional units, including BSH activity, *bai*-gene modules, bile-acid ratios, receptor-response signatures and microbial-metabolic phenotypes. A good biomarker should be reproducible and interpretable across dietary, co-medication, disease activity and sampling compartment. Beyond taxonomy, functional metagenomics, targeted bile-acid profiling of the stool and plasma, and measuring host-signaling, ideally over time, can give a more solid basis (Vich Vila et al., 2024).

Translation remains limited because much TCM evidence comes from bile-acid chelator, high-fat-diet, and chemical-colitis models that do not capture clinical exposure histories, comorbidities, polypharmacy, or heterogeneity. Biomarker improvement is not validated clinical benefit, particularly in progressive liver disease or IBD. Product variability, chemical quality control, herbal-drug interactions, clinical endpoints, and safety monitoring must accompany the proposed mechanism to support therapeutic inference (Liu Q. et al., 2024).

This article is a structured narrative review rather than a systematic review, scoping review, or meta-analysis. It examines TCM modulation of the gut microbiota-bile acid axis in MASLD, obesity/T2D, and IBD and organizes primary evidence around a putative pathway from intervention standardization to microbial function, bile-acid species, concentrations or profiles, receptor and cellular responses, and clinical or preclinical outcomes. This framework enables comparison of heterogeneous studies and distinguishes reproducible, pathway-linked findings from associations. The review does not aim to catalogue every herbal medicine reported to affect the microbiota; it evaluates whether reported effects are specific, reproducible, and sufficiently documented to warrant further development. The search, screening, appraisal, and synthesis procedures are described below to make the narrative review transparent and reproducible.

### 1.1 Structured narrative review methodology and study appraisal

We conducted a structured literature search to identify primary studies on TCM, the gut microbiota-bile acid axis, and metabolic or inflammatory diseases. PubMed/MEDLINE, Embase, Web of Science Core Collection, and Scopus were searched from database inception to 25 August 2025; the final search was run on 25 August 2025. Search concepts were combined with Boolean operators and adapted to each database: (traditional Chinese medicine OR TCM OR herbal medicine OR botanical OR formula OR phytochemical) AND (gut microbiota OR microbiome OR microbial OR dysbiosis) AND (bile acid* OR bile salt* OR FXR OR NR1H4 OR TGR5 OR GPBAR1) AND (metabolic dysfunction-associated steatotic liver disease OR MASLD OR non-alcoholic fatty liver disease OR NAFLD OR obesity OR type 2 diabetes OR inflammatory bowel disease OR IBD OR colitis). Reference lists of relevant reviews and included articles were hand-searched for additional studies. The databases, search date, search concepts, and citation-chaining procedure are reported to support reproducibility. Because this is a structured narrative review, PRISMA 2020 is not claimed as the reporting standard and no quantitative synthesis was planned. NAFLD was retained in the search string because it is the historical term used by earlier studies; it was not treated as interchangeable with MASLD in the synthesis. Original article titles retain their source terminology in the reference list.

Eligible primary publications were English-language, peer-reviewed original research in humans, relevant animal models, or mechanistic in-vitro systems that (i) evaluated a defined TCM preparation, constituent, or clinically used formula; (ii) reported at least one microbiota-related, bile-acid, receptor/signalling, or disease-relevant outcome; and (iii) provided sufficient information on the intervention and comparator. Relevant reviews were used for contextual mapping and backward citation searching but were not treated as primary evidence. We excluded editorials, conference abstracts, case reports, studies without an identifiable TCM intervention, and papers focused solely on unrelated microbiome or bile-acid outcomes. Two authors independently screened titles and abstracts, retrieved potentially relevant full texts, and recorded reasons for exclusion; disagreements were resolved through discussion with a third author. Because interventions, models, endpoints, and sampling compartments were heterogeneous, findings were synthesized narratively rather than pooled quantitatively.

Study quality and risk of bias were assessed independently by the same two reviewers using design-appropriate tools: RoB 2 for randomized clinical trials (Sterne et al., 2019), ROBINS-I for non-randomized intervention studies (Sterne et al., 2016), SYRCLE risk-of-bias tool for animal studies (Hooijmans et al., 2014). Reviews were not treated as primary evidence or included in intervention-level evidence grades. Domains considered included randomization and allocation concealment, deviations from intended interventions, missing data, outcome measurement, selective reporting, confounding, and conflicts of interest. Judgements were classified as low, some concerns, or high risk (or the corresponding tool-specific categories) and were used to weight the strength of mechanistic and translational conclusions. Across the included intervention studies, the Qushi Huayu randomized trial raised some concerns because microbiota and bile-acid endpoints were incomplete. Most animal studies were judged at unclear risk because randomization, allocation concealment, blinding, attrition, dose reporting, or formula characterization were insufficiently reported. These judgements were incorporated into the evidence grades: animal studies remained Level C unless pathway-specific causal validation was reported, and microbiota-associated findings without pathway interruption remained Level D for mediation. Study-level judgements, key domains, and their implications for the evidence grades are reported in Table 1.

**Table 1:** Study-level risk-of-bias assessment for TCM intervention studies used in the evidence-grading framework.

Study	Design/model	Tool	Overall judgement	Key domains contributing to judgement	Implication for evidence grade
Qushi Huayu (Liu Q. et al., 2024)	Randomized clinical trial; 246 adults enrolled under the historical NAFLD designation	RoB 2	Some concerns	Axis-related microbiota outcomes were reported, but serial bile-acid endpoints were incomplete for mediation assessment.	Level A for clinical efficacy; Level D for TCM-microbiota-bile-acid mediation.
JiGuCao capsule (Qi et al., 2025)	HFD-induced mouse model	SYRCLE	Unclear risk	Randomization, allocation concealment, blinding, attrition, and intervention characterization were incompletely reported.	Level C; coordinated preclinical evidence without pathway-specific validation.
Jiang-Tang-San-Huang pill (Tawulie et al., 2023)	HFD/STZ-induced rat model of T2D	SYRCLE	Unclear risk	Randomization, allocation concealment, blinding, attrition, and outcome-reporting procedures were incompletely reported.	Level C; no pathway-specific perturbation or mediation test.
Shengmai San (Wang Z. et al., 2024)	HFD-induced mouse model of obesity	SYRCLE	Unclear risk	Randomization, allocation concealment, blinding, attrition, dose reporting, and formula characterization were incompletely reported.	Level C; antibiotic depletion supported microbiota dependence and FMT supported transferability, but bile-acid-specific mediation was untested.
Modified Gegen Qinlian decoction (Liu R. et al., 2024)	db/db mouse model of T2D	SYRCLE	Unclear risk	Randomization, allocation concealment, blinding, attrition, dose reporting, and formula characterization were incompletely reported.	Level C; coordinated response without pathway-specific validation.
Banxia Xiexin decoction (Luo et al., 2024)	DSS-induced mouse model of colitis	SYRCLE	Unclear risk	Randomization, allocation concealment, blinding, attrition, dose reporting, and formula characterization were incompletely reported.	Level C for colitis outcomes; Level D for bile-acid mediation.
Gegen Qinlian decoction (Wang X. et al., 2023)	DSS-induced mouse model of colitis	SYRCLE	Unclear risk	Randomization, allocation concealment, blinding, attrition, dose reporting, and formula characterization were incompletely reported.	Level C for colitis outcomes; Level D for bile-acid mediation.

Judgements were based on reported study methods and outcomes. Unclear risk denotes insufficient reporting for at least one critical design or reporting domain. Evidence grades assess the strength of intervention-to-pathway inference and are not risk-of-bias ratings.

### 1.2 Distinguishing TCM-specific evidence from general biological context

General literature on gut microbiota, bile-acid metabolism, FXR/TGR5 signalling, and microbiome biology is used in this review to establish biological context and to define measurable pathway components; it is not treated as evidence that TCM produces those effects. TCM-specific evidence is restricted to studies in which a named TCM formula, botanical preparation, purified TCM constituent, or TCM-derived microbial metabolite is the experimental exposure and the reported microbiota, bile-acid, receptor, or disease outcome is measured in the same study. Accordingly, mechanistic principles from generic reviews (for example, bile-salt hydrolase activity, *bai*-gene pathways, or FXR/TGR5 biology) are presented as hypotheses or interpretive benchmarks, whereas intervention-specific findings are identified with the relevant TCM citation.

In the TCM evidence sections, we therefore separate three levels of support: (i) TCM intervention evidence, comprising data generated after administration of a defined formula or constituent (for example, altered bile-acid metabolism or TGR5-associated signalling reported for Jiang-Tang-San-Huang pill, modified Gegen Qinlian decoction, Shengmai San, ZeXieYin, and related preparations); (ii) pathway evidence, in which microbial functions, bile-acid species, or FXR/TGR5 responses are directly measured and linked to the TCM exposure; and (iii) causal evidence, which is subdivided into microbiota-dependence evidence and pathway-specific mediation evidence. Microbiota depletion tests whether an effect depends on an intact microbiota, whereas FMT tests whether a phenotype or treatment response is transferable; both interventions also produce broad physiological, metabolic, and immunological changes in the host. Neither result identifies a specific bile-acid transformation as the mediator. We reserve pathway-specific mediation for studies that measure the candidate microbial function and bile-acid intermediate in a relevant compartment and add a pathway-directed test, such as exposure-matched metabolite add-back, defined-strain or gene manipulation, isotope tracing, enzyme inhibition, or receptor perturbation, that changes or rescues the phenotype. Studies that only describe generic microbiome biology or a disease-associated bile-acid pattern are retained as context and are not counted as TCM efficacy or mechanism evidence. This distinction is applied throughout Sections 2–6 and in Tables 2–4.

**Table 2:** Microbial bile-acid transformations relevant to the TCM–gut microbiota–bile-acid axis, with study model, dose, safety-reporting status and evidentiary boundary.

Microbial function/representative study	Study model/population; TCM exposure and dose	Major microbiota and bile-acid finding	Mechanistic readout/host node	Evidence grade	Safety outcome/reporting; critical limitation
BSH deconjugation; JiGuCao capsule (Qi et al., 2025)	HFD-induced mice; JiGuCao capsule (animal dose reported by study; standardized extract required)	BSH-capable taxa and conjugated/unconjugated BA ratios changed; species-level taxa were study-specific	Altered intestinal BA pool may influence FXR-FGF15 and hepatic lipid handling	C (coordinated preclinical evidence)	Safety: adverse events, liver/renal toxicity and herb-drug interactions were not reported in the indexed preclinical record. Limitation: no microbiota depletion or metabolite add-back; dose/exposure is not comparable across batches.
BSH-linked deconjugation; Jiang-Tang-San-Huang pill (Tawulie et al., 2023)	Male HFD/STZ-induced T2D rats; oral 0.27, 0.54 or 1.08 g kg^−1^for 4 weeks; metformin control.	*Bacteroides*, *Lactobacillus* and *Bifidobacterium* with reported BSH activity increased; ileal unconjugated CDCA and DCA increased. *Bai* genes were not measured, and the reported ileal bile-acid concentrations do not establish enterohepatic flux.	Associations with intestinal FXR/FGF15 and TGR5/GLP-1 signalling, together with hepatic CYP7A1/CYP8B1 readouts.	C (coordinated preclinical response)	Safety: adverse events, organ toxicity and herb-drug interactions were not reported in the indexed preclinical record. Limitation: no depletion/FMT, metabolite add-back or receptor perturbation; BSH capacity and bile-acid concentrations do not establish enterohepatic flux.
Secondary BA production; Shengmai San (Wang Z. et al., 2024)	HFD-induced diet-induced-obesity mice; oral Shengmai San, dose-dependent regimen (numeric dose and duration were not available in the indexed abstract).	*Lactobacillus* carrying BSH enzymes decreased; conjugated bile acids, including TCA, increased in serum, ileum and inguinal white adipose tissue.	TCA was associated with M2-macrophage Slit3 release and sympathetic PKA/CaMKII signalling that promoted adipose browning.	C (antibiotic depletion supported microbiota dependence; FMT supported transferability; bile-acid-specific mediation untested)	Safety: adverse events, organ toxicity and interaction endpoints were not reported in the indexed preclinical record. Limitation: no exposure-matched TCA add-back or receptor blockade; rodent formulation/dose cannot be extrapolated to humans.
Oxidation/epimerization; TUDCA intervention (Wang H. et al., 2024)	Experimental mice from a study using the historical NAFLD label	TUDCA reshaped BA metabolism and gut microbial profile	Barrier and hepatic metabolic effects involving BA signaling	C	Safety: not a TCM formula study; formal safety and interaction assessment is outside the mechanistic question. Limitation: TUDCA has direct host actions, so microbiota-mediated inference remains unresolved.
Microbial BA–host validation (Makki et al., 2023; Nie et al., 2024)	Fiber-fed mice / MASH models; no TCM exposure (mechanistic comparator)	6α-hydroxylated or secondary BAs linked to TGR5 signaling; defined gut symbionts alleviated MASH	TGR5 or secondary-BA biosynthetic pathway altered glucose or liver inflammation	B (pathway perturbation in non-TCM models)	Safety: not applicable to these non-TCM mechanistic comparators. Limitation: pathway plausibility does not establish a TCM-specific effect, safety profile or clinical benefit.

**Table 3:** Representative TCM interventions targeting the axis: study models/populations, dose, microbiota–bile-acid findings, mechanism, safety-reporting status, evidence grade and translational needs.

TCM therapy (representative study)	Study model/population and dose	Major microbiota/bile-acid finding	Mechanistic readout	Evidence grade	Safety outcome/reporting; key limitation/next validation
Qushi Huayu (Liu Q. et al., 2024)	Adults enrolled under the historical NAFLD designation, *n* = 246; oral QSHY versus DFLG, 24 weeks; fixed clinical doses, not body-weight-normalized.	Clinical outcomes and no new serious safety signal reported; microbiota associations identified, but serial BA profiling was not prespecified	ALT, AST and FIB-4 improved; *Escherichia–Shigella*, *Bacteroides* and aromatic amino-acid metabolites associated with ALT response	A for clinical efficacy; D for TCM–microbiota–BA mediation	Safety: no new serious safety signal was reported over 24 weeks. Limitation: adverse-event detail, interaction adjudication and serial laboratory safety reporting do not establish safety across doses, batches or co-medications; add serial stool/plasma bile acids, functional metagenomics and mediation analysis.
Modified Gegen Qinlian decoction (Liu R. et al., 2024)	db/db T2D mice; oral modified Gegen Qinlian decoction, high-, medium- and low-dose groups for 12 weeks (numeric g kg^−1^doses not reported in the indexed article).	Community and BA-metabolism shifts identified by metagenomics/targeted metabolomics	TGR5-associated signaling and glycaemic improvement	C	Safety: adverse events, organ toxicity and herb-drug interactions were not reported in the indexed preclinical record. Limitation: no depletion/FMT or receptor perturbation; validate dose–exposure and the causal chain in humans.
Jiang-Tang-San-Huang pill (Tawulie et al., 2023)	Male HFD/STZ-induced T2D rats; oral 0.27, 0.54 or 1.08 g kg^−1^for 4 weeks; metformin control.	BSH-associated taxa increased and ileal unconjugated CDCA/DCA increased; *bai* genes were not measured, and the reported ileal bile-acid concentrations do not establish enterohepatic flux.	Associations with intestinal FXR/FGF15 and TGR5/GLP-1 signalling.	C	Safety: adverse events, organ toxicity and interaction outcomes were not reported in the indexed preclinical record. Limitation: taxonomic association and batch variability; test active fractions, exposure-matched metabolite add-back and receptor dependence.
Shengmai San (Wang Z. et al., 2024)	HFD-induced diet-induced-obesity mice; oral Shengmai San, dose-dependent regimen (numeric dose and duration were not available in the indexed abstract).	Decreased BSH-carrying *Lactobacillus* and increased conjugated bile acids, including TCA, across serum, ileum and adipose tissue.	TCA-associated M2 macrophage–Slit3 and sympathetic thermogenic programme; antibiotic depletion supported microbiota dependence and FMT supported transferability.	C (microbiota dependence and transferability supported; BA-specific mediation untested)	Safety: adverse events, organ toxicity and interaction outcomes were not reported in the indexed preclinical record. Limitation: test defined metabolites, exposure-matched add-back, sex effects and human pharmacology.
Banxia Xiexin / Gegen Qinlian decoctions (Luo et al., 2024; Wang X. et al., 2023)	DSS-colitis mice; oral decoctions, study-specific g kg^−1^regimens (numeric doses not consistently available in indexed records).	Microbiota and microbial metabolism changed; BA axis not fully resolved	Barrier repair and immune-metabolic signaling (AhR/IL-22 in Gegen Qinlian)	C for colitis outcomes; D for BA mediation	Safety: adverse events, organ toxicity and interaction outcomes were not reported in the indexed preclinical records. Limitation: targeted bile-acid panels, mucosal exposure measurements and receptor-proximal perturbation are needed.

**Table 4:** Characterisation, exposure and safety/interaction reporting status of representative TCM interventions discussed in this review.

Intervention (study)	Formula identity/botanical authentication	Formulation/extraction	Chemical markers/fingerprint	Dose, route, duration	Lot/quality control	Reporting status, safety/interaction reporting and inference boundary	Characterisation tier and permissible inference
Qushi Huayu (Liu Q. et al., 2024)	Empirical formula (QSHY); individual botanical components and voucher information not reported in indexed record.	Oral clinical product; extraction process and final dosage form not reported in indexed record.	Not reported in indexed record.	Oral QSHY versus DFLG control; 24 weeks; dose description and daily amount not reported in indexed record.	Manufacturer, lot, inter-batch comparison, and release QC not reported in indexed record.	Clinical evidence applies to the tested QSHY product. Safety: no new serious safety signal was reported over 24 weeks, but interaction adjudication and cross-batch safety cannot be assessed from the indexed record.	Tier III. The accessible record does not permit confirmation of authentication, chemical markers, or lot comparability. Clinical findings apply to the trial product/batch only.
Modified Gegen Qinlian decoction (Liu R. et al., 2024)	Traditional Gegen Qinlian decoction modified by addition of dried ginger; full composition, plant parts, and vouchers not reported in indexed record.	Oral decoction; extraction conditions and yield not reported in indexed record.	Not reported in indexed record.	db/db mice; high-, medium-, and low-dose groups; 12 weeks; numeric doses not reported in indexed record.	Not reported in indexed record.	Biological result is product-specific; chemical comparability cannot be assessed. Safety/interactions: not reported in the indexed preclinical record.	Tier III. The accessible record does not establish a reproducible product identity or chemical equivalence. Preclinical findings apply to the administered decoction only.
Jiang-Tang-San-Huang pill (Tawulie et al., 2023)	Named TCM prescription; botanical composition and vouchers not reported in indexed record.	Oral pill; manufacturing and extraction details not reported in indexed record.	Not reported for administered pill; UPLC-MS/MS was used for ileal bile-acid profiling, not product QC.	Male SD rats; oral 0.27, 0.54, and 1.08 g kg^−1^; 4 weeks.	Lot, inter-batch comparison, and release QC not reported in indexed record.	Dose is defined, but product-specific identity and batch equivalence cannot be assessed. Safety/interactions: not reported in the indexed preclinical record.	Tier III. Defined dose does not establish product identity or chemical equivalence. Findings apply to the administered pill/batch only.
Shengmai San (Wang Z. et al., 2024)	SMS formula; botanical composition and vouchers not reported in indexed record.	Oral formula; preparation and extraction not reported in indexed record.	Not reported in indexed record.	HFD-induced mice; dose-dependent oral treatment; numeric dose and duration not reported in indexed record.	Not reported in indexed record.	Preclinical result applies to the tested formulation; composition comparability is not assessable. Safety/interactions: not reported in the indexed preclinical record.	Tier III. The accessible record does not establish composition comparability. The phenotype applies to the administered formulation/batch only.
Banxia Xiexin decoction (Luo et al., 2024)	Classical BXD prescription; botanical composition and vouchers not reported in indexed record.	Preparation and extraction not reported in indexed record.	Major constituents identified by HPLC-DAD; marker identities and acceptance criteria not stated in indexed record.	Male C57BL/6 mice; high-, medium-, and low-dose groups; numeric dose and duration not reported in indexed record.	Lot and QC information not reported in indexed record.	HPLC-DAD supports some profiling, but authentication and batch control remain unassessable. Safety/interactions: not reported in the indexed preclinical record.	Tier II. HPLC-DAD provides partial chemical profiling, but authentication and lot-level acceptance criteria remain unassessable. Findings apply to the described preparation only.
Gegen Qinlian decoction (Wang X. et al., 2023)	Traditional formula attributed to Treatise on Febrile Diseases; botanical details and vouchers not reported in indexed record.	Preparation and extraction not reported in indexed record.	Not reported in indexed record.	DSS colitis model (3% DSS for 6 days); GQD dose, route, and duration not reported in indexed record.	Not reported in indexed record.	Mechanistic result is product-specific; chemical definition and batch equivalence cannot be assessed. Safety/interactions: not reported in the indexed preclinical record.	Tier III. The accessible record does not establish chemical definition or batch equivalence. Mechanistic findings apply to the tested decoction/batch only.

## 2 The gut microbiota-bile acid axis: microbial metabolism, host signaling, and causal interpretation

### 2.1 Microbial remodeling of the bile-acid pool

The liver synthesizes and conjugates primary bile acids, which enter the intestine and undergo distal microbial transformation. BSHs deconjugate bile salts; *bai* genes mediate 7alpha-dehydroxylation; hydroxysteroid dehydrogenases diversify the pool through hydroxylation and epimerization; and microbial acyltransferases form amino-acid conjugates (Guzior and Quinn, 2021). Gene and strain information should be integrated with enzyme activity and metabolite concentrations rather than taxonomic abundance alone. Bile acids also act as endocrine and paracrine signals in multiple organs (Perino et al., 2021; Guzior et al., 2024).

Bile acids impose detergent and membrane stress on microbes, whereas microbes determine the bile acids available to epithelial and systemic receptors. Diet, transit, inflammation, host genotype, hepatic synthesis, bile-acid sequestrants, antibiotics, metformin, and surgery modify this feedback loop. A taxon-bile-acid association cannot identify the producer, establish altered enterohepatic flux, or exclude host selection. When BSH or *bai* activity is proposed, functional metagenomics with targeted stool and serum bile-acid measurements is more informative than 16S rRNA sequencing alone (Foley et al., 2021).

Gene abundance indicates transformation capacity, not gene expression, enzyme activity, substrate availability, or net enterohepatic flux; related strains can also differ in BSH, *bai*, and hydroxysteroid dehydrogenase genes. Metatranscriptomics, targeted enzyme assays, cultivation, and isotope-based approaches strengthen metagenomic evidence. Stool, intestinal content, portal blood, systemic blood, and tissue samples capture distinct stages of enterohepatic cycling, particularly relevant when TCM alters diet, transit, or host secretion (Guzior and Quinn, 2021; Collins et al., 2023; Ding et al., 2025).

Bile-acid transformations complicate taxonomic interpretation: bile acids can inhibit susceptible organisms, select resistant populations, and alter pathway expression. A change in a putatively beneficial taxon may thus follow, rather than cause, an altered bile-acid environment; a low-abundance organism may still carry the relevant active pathway. Studies should report the compartment, sampling time, and exposure conditions, and test whether a defined activity accounts for the measured bile-acid profile rather than whether a familiar genus changed (Collins et al., 2023; Guzior and Quinn, 2021).

The microbial bile-acid repertoire should be interpreted with other microbiota-derived metabolites rather than as an isolated signal (Fogelson et al., 2023). In a human-to-mouse study of cholesterol gallstone disease, microbiota-associated changes in secondary bile acids and bile-acid hydrophobicity accompanied greater intestinal cholesterol absorption and gallstone formation (Hu et al., 2022). The consequences of a bile-acid shift therefore depend on the disease setting and the outcome being measured (Cai et al., 2022b).

### 2.2 Host responses to microbial bile-acid metabolism

FXR and TGR5 have tissue-, bile-acid-, and disease-specific roles. Intestinal FXR controls FGF15/19 signalling and negative feedback to hepatic bile-acid synthesis, whereas TGR5 regulates GLP-1 secretion, energy expenditure, macrophage polarization, and inflammatory signalling. Bile acids also affect barrier programs, inflammasome activity, innate immunity, and T-cell differentiation through receptor-dependent and independent mechanisms (Mohanty et al., 2024). Receptor mRNA alone is insufficient: studies should establish downstream signalling, target cells, and its relation to disease improvement (Zhang S. Y. et al., 2021; Chiang and Ferrell, 2022).

Where mechanistic claims are made, we now cite original intervention or pathway-perturbation studies whenever available. For example, Makki et al. (2023) experimentally linked 6α-hydroxylated bile acids to TGR5-dependent improvements in glucose metabolism in mice; Nie et al. (2024) identified a gut-symbiont secondary-bile-acid biosynthetic pathway that attenuated MASH; Wang Q. et al. (2023) demonstrated microbiota regulation of postprandial GLP-1 through ileal bile acid–TGR5 signaling; Zheng et al. (2024) linked ileitis-associated bile-acid changes to TGR5 signaling in ileal CD8^+^T cells; and Zhang S. Y. et al. (2021) used central FXR perturbation to test microbial bile-acid effects on insulin action. These primary studies support pathway plausibility in defined models but do not establish that an individual TCM formula acts through the same mechanism.

The variety of immune effects of bile-acid-regulated receptors can be attributed to the presence of these receptors on monocytes, macrophages, dendritic cells, lymphoid cells, and T-cell subsets (Fiorucci et al., 2024). Although bile acids have been associated with the activity of gut microbes and systemic immunometabolism, their association does not define the target cell, ligand or receptor in a given intervention (Guan et al., 2022; Li et al., 2021; Cai et al., 2022a).

FXR and TGR5 effects are tissue, bile-acid composition and disease-state dependent. Intestinal FXR controls FGF15/19 and hepatic bile-acid formation, and TGR5 controls GLP-1, energy expenditure, macrophage polarization and inflammatory signaling. Bile acids also have an impact on barrier, inflammasome, innate immune, and T-cell programs. Studies should link receptor transcript expression to downstream signaling events, target cells, and disease outcomes since receptor transcripts alone do not provide a mechanism (Mohanty et al., 2024; Lee et al., 2024).

### 2.3 Interpreting causal evidence along the gut microbiota-bile acid axis

We applied prespecified, mutually exclusive criteria and assigned each study the highest level whose mandatory requirements it met. Level A required a prospective controlled human study or well-characterized longitudinal cohort with standardized TCM exposure and an appropriate comparator. It additionally required at least two sampling points, function-resolved microbiome analysis, quantitative bile-acid profiling in a relevant compartment, a receptor-proximal or host-response readout, a disease-relevant clinical outcome, and pathway-specific support. Depletion established microbiota dependence and FMT established transferability, not mediation by a specific bile-acid transformation; mediation required direct measurement of the candidate function and intermediate plus metabolite add-back, defined-strain or gene manipulation, isotope tracing, enzyme inhibition, or receptor perturbation. Independent human replication required temporal and functional support. Level B required a controlled animal intervention with standardized exposure and comparator, longitudinal assessment of at least two pathway layers, including microbial function, bile acids, host signalling, or disease outcome, and a perturbation that changed or rescued the phenotype. Level C comprised controlled human, animal, or in-vitro studies with coordinated intervention, microbiota, bile-acid, and host or disease responses but without Level A/B validation; depletion or FMT alone remained Level C. Level D comprised cross-sectional, uncontrolled, association-only, or inadequately characterized studies. Studies were assigned the highest level only when all mandatory criteria were documented. This hierarchy preserves discovery evidence while limiting unsupported mechanistic claims. Figure 1 operationalizes the hierarchy across intervention standardization, microbial function, bile-acid exposure, host responses, outcomes, and contextual modifiers.

**Figure 1:**
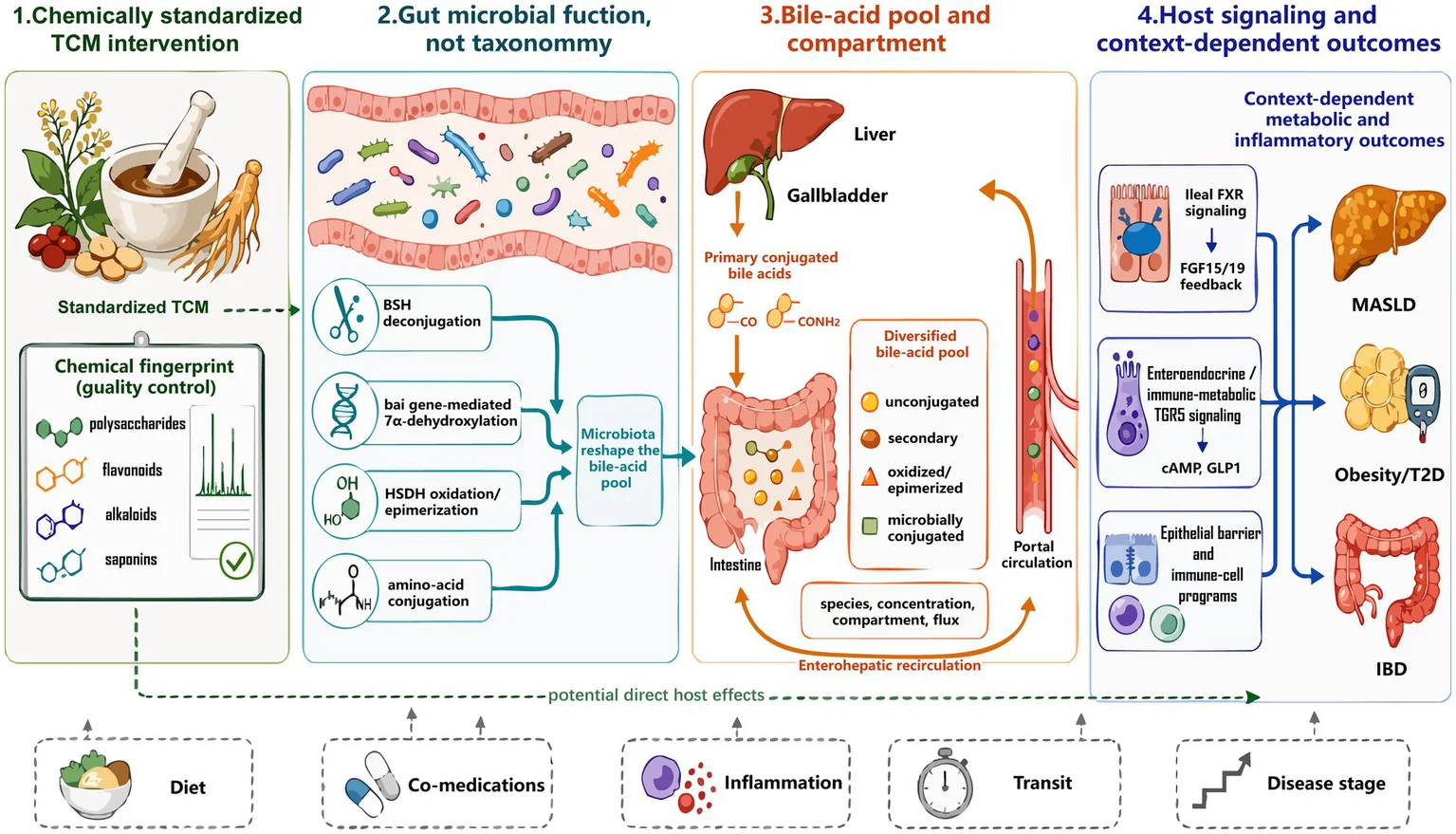
Conceptual framework for evaluating traditional Chinese medicine (TCM) modulation of the gut microbiota-bile acid axis in metabolic and inflammatory diseases. The framework proceeds from a chemically standardized TCM intervention (authentication, extraction, marker compounds and batch quality control) to microbial functions rather than taxonomy alone, including bile salt hydrolase (BSH) deconjugation, *bai* gene-mediated 7α-dehydroxylation, hydroxysteroid dehydrogenase (HSDH) oxidation/epimerization and microbial amino-acid conjugation. These functions reshape the bile-acid pool across intestinal, portal and systemic compartments through enterohepatic circulation. The resulting bile-acid species, concentrations and compartment-specific exposure can engage intestinal FXR-FGF15/19 feedback, enteroendocrine and immune-metabolic TGR5 signaling, epithelial-barrier programs and immune-cell pathways, producing context-dependent outcomes in MASLD, obesity/T2D and IBD. Direct host effects of TCM and major modifiers (diet, co-medications, inflammation, intestinal transit and disease stage) are shown as parallel influences. The figure is a conceptual model: coordinated changes do not establish mediation without functional and pathway-directed validation.

Causal inference requires measurement of the proposed bile-acid intermediate in the relevant compartment and temporal sequence. Stool reflects distal luminal exposure; plasma, portal blood, and tissue address distinct questions. Circulating bile acids do not directly establish tissue-specific FXR or TGR5 activation because local transport, uptake, protein binding, intracellular conversion, and receptor abundance can decouple plasma concentrations from ligand exposure at the target site. Tissue or receptor-proximal measurement is required for mechanisms in the intestine, liver, adipose tissue, or immune cells (Zhang S. Y. et al., 2021; Wang Q. et al., 2023). Studies should quantify substrates and products, distinguish microbial capacity from expression and activity, measure enterohepatic flux when claimed, and test effects on receptor-proximal and disease outcomes (Collins et al., 2023; Guzior and Quinn, 2021). Table 2 lists major transformations, relevant consequences, and host signalling nodes.

Causal inference should be designed before treatment. The proposed pathway should specify expected changes in exposure, microbial function, bile-acid intermediates, host response, and outcome, thereby defining comparators and distinguishing mediators from biomarkers. Repeated sampling establishes temporal order, and chemically and analytically interpretable negative results can identify a broken link in the chain (Figure 2).

**Figure 2:**
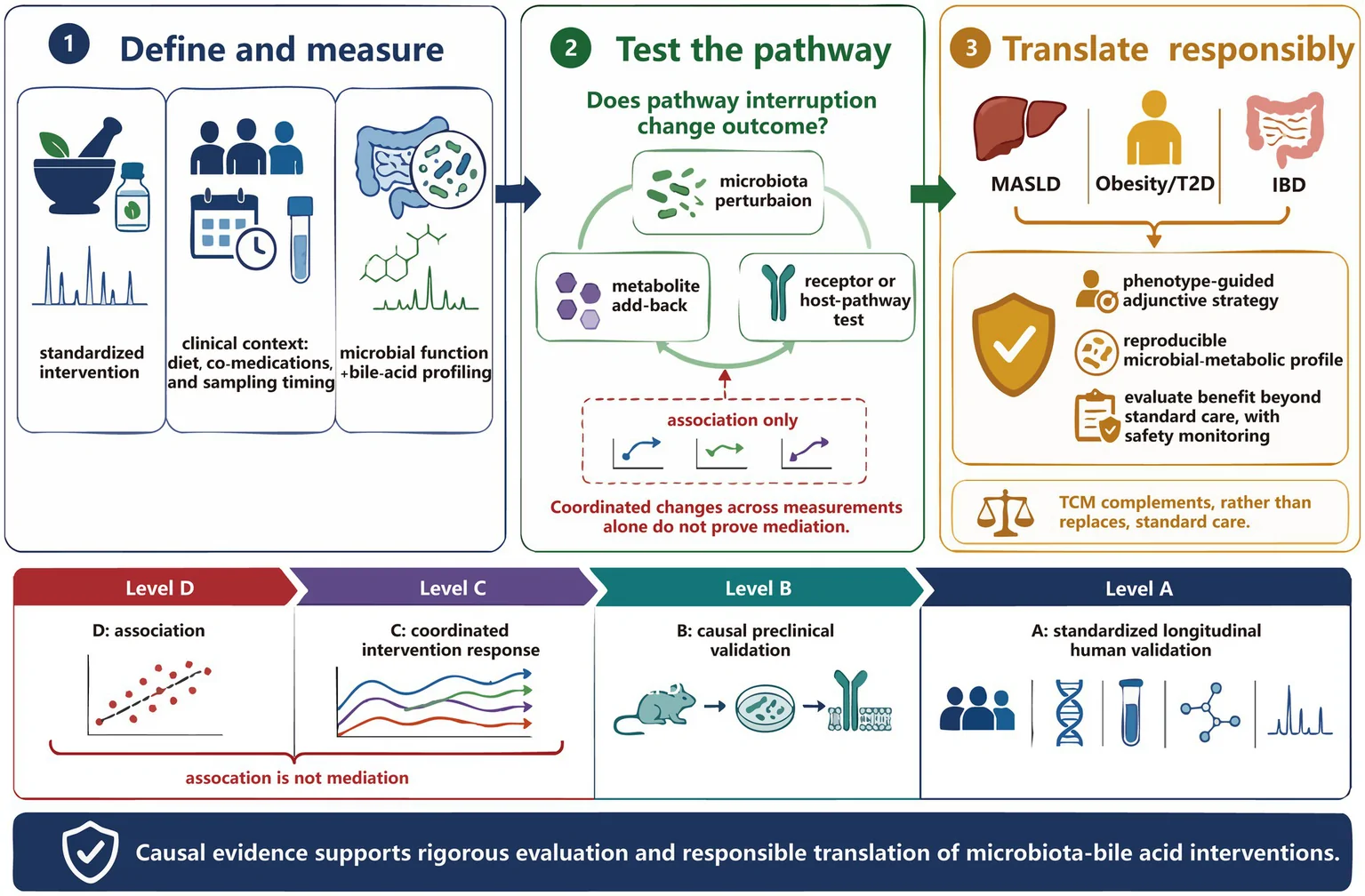
Evidence framework for causal evaluation and responsible translation of TCM interventions targeting the gut microbiota–bile acid axis. Step 1 defines the standardized intervention, clinical context, microbial function and bile-acid profile. Step 2 tests the pathway sequentially: microbiota depletion assesses dependence and FMT assesses transferability, whereas exposure-matched metabolite add-back, defined-strain or gene manipulation, isotope tracing, enzyme inhibition, or receptor/host-pathway testing is required to support pathway-specific mediation. Depletion or FMT alone does not constitute pathway-specific causal evidence. Step 3 translates validated signals in MASLD, obesity/T2D and IBD through phenotype-guided adjunctive strategies, reproducible microbial–metabolic profiles, benefit beyond standard care and safety monitoring. The evidence scale comprises Level D (association), Level C (coordinated response or microbiota dependence without pathway-specific interruption), Level B (causal preclinical validation) and Level A (standardized longitudinal human validation with functional, clinical and pathway-specific evidence). Progression requires increasingly direct evidence; not every intervention will reach Level A.

## 3 TCM-mediated modulation of the gut microbiota-bile acid axis

We extracted human clinical evidence using a prespecified template covering study design, population, sample size, intervention and comparator, dose, duration, clinical outcomes, microbiota and bile-acid measurements, and safety. Only one eligible TCM trial reported both clinical outcomes and an axis-related microbial endpoint: the Qushi Huayu study in adults enrolled under the historical NAFLD designation (Liu Q. et al., 2024). No eligible human comparative study in obesity/T2D or IBD reported a defined TCM exposure together with microbiota–bile-acid measurements. Those literatures therefore remain preclinical or observational in this review.

In this multicentre, double-blind, double-dummy trial, 246 adults with liver dysfunction, enrolled under the study’s historical NAFLD designation, were randomized 1:1 to oral QSHY plus a Dangfei Liganning (DFLG) simulant or QSHY simulant plus DFLG for 24 weeks. The protocol used fixed clinical doses for both products; the study did not express dose as body-weight-normalized exposure. Primary outcomes were changes in serum alanine aminotransferase (ALT) and liver controlled attenuation parameter (CAP) measured by vibration-controlled transient elastography. MRI-proton density fat fraction (MRI-PDFF) was assessed as a secondary outcome in a 79-participant single-centre subset.

Both groups improved liver fat and enzyme concentrations. Compared with DFLG, QSHY reduced ALT (*β* = −0.128, 95% CI − 0.25 to −0.005, *p* = 0.041), AST (*β* = −0.134, 95% CI − 0.256 to −0.012, *p* = 0.032), and FIB-4 (*β* = −0.129, 95% CI − 0.254 to −0.003, *p* = 0.044), whereas between-group differences in CAP (*p* = 0.503) and MRI-PDFF (*p* = 0.408) were not significant. Changes in *Escherichia–Shigella* and *Bacteroides* abundance, serum phenylalanine and tyrosine, and microbial p-hydroxyphenylacetic acid were associated with ALT response. The trial reported no new serious safety signal. However, serial targeted bile-acid profiling, receptor-proximal readouts, and pathway perturbation were not prespecified, so these associations do not establish a TCM-specific bile-acid mechanism or safety across other doses, products, or co-medications.

Accordingly, the Qushi Huayu trial supports Level A evidence for clinical efficacy but Level D evidence for TCM–microbiota–bile-acid mediation, whereas the preclinical studies remain below the threshold for clinical validation. The evidence map in Figure 1 links TCM exposure, microbial function, bile-acid measurements, host responses and disease outcomes; the detailed causal criteria are defined in Section 2.3.

### 3.1 Chemical standardization and microbial transformation of TCM

TCM interventions are not single compounds, but chemically complex mixtures. The constituents reaching the intestine and the substrates upon which microbes can act are dependent on botanical authentication, chemical fingerprint, extraction procedure, dose and batch consistency. Polysaccharides can alter the availability of substrates and the ecology of the microorganisms. Flavonoids, glycosides, saponins may undergo deglycosylation and other modifications and microbial products may be different from the parent compounds in absorption or receptor activity. This complexity creates therapeutic opportunities but also complicates reproducibility. Before assigning a microbial mechanism, studies should report voucher information, quantitative marker compounds, extraction procedures, stability, and contaminant testing (Zhang H. Y. et al., 2021; Li et al., 2024b; Hao et al., 2025; Wei et al., 2024). Delivery design is an additional source of exposure variation. Nanomedicine-based strategies can modify the release, tissue distribution and bioavailability of natural products, and should therefore be characterized as part of the intervention rather than treated as neutral formulation detail (Zeng et al., 2023).

To evaluate TCM interventions consistently, we expanded the extraction template to six mandatory domains: (1) identity of the administered material, including the accepted Latin binomial, plant part, formula composition and proportion, manufacturer or source, and voucher specimen or authentication method; (2) preparation and manufacturing, including processing, extraction solvent, solvent-to-material ratio, temperature/time when available, extraction yield, excipients, and final dosage form; (3) chemical characterization, including the analytical platform, lot-specific chromatographic or spectrometric fingerprint, quantitative marker compounds, reference standards, and stated acceptance limits; (4) exposure, including route, amount per administration and per day, dose rationale, frequency, duration, and timing relative to diet and biological sampling; (5) batch and quality controls, including batch number, between-batch comparability, stability, and testing for contaminants, adulterants, pesticides, heavy metals, and microbial burden; and (6) experimental reproducibility, including sufficient reporting to reproduce the administered product and its exposure. Each domain was coded as adequately reported, partially reported, or not reported. A study was considered chemically reproducible only when the administered material, preparation method, dose/exposure, and at least one identity- or quantity-based analytical measure were adequately documented.

For multi-herb formulae, we additionally distinguished the raw-herb prescription from the final administered product and recorded each ingredient at species and plant-part level, its amount or proportion, processing or substitution, and the manufacturer or pharmacy responsible for dispensing. For proprietary products, a product name alone was not considered sufficient: the source, dosage form, lot identifier, and available certificate or release specification were recorded. We reported human exposure as daily amount, route, frequency, and duration; animal exposure as g kg^−1^ day^−1^(or the original unit) together with the formulation administered. Raw-herb-to-extract conversions were not inferred when the extraction yield was not reported.

Batch-to-batch consistency was assessed separately from a nominal fingerprint: an adequately documented comparison required lot-specific analytical data for the same markers and fingerprint, together with the stated acceptance range or a justified similarity criterion. We did not infer chemical equivalence from a common formula name, and we did not convert an incompletely described decoction into an assumed extract dose. When botanical authentication, formulation, extraction, chemical markers, dose, lot information, or quality-control data were absent, the domain was designated as not reported; the associated biological result was consequently interpreted as evidence for the tested preparation or batch, rather than for the named formula in general. This conservative approach is consistent with best-practice guidance for phytopharmacological research (Heinrich et al., 2020).

Additional requirements were applied to complex multi-herb formulae. Formula composition had to be reported at the species and plant-part level together with the amount or proportion of each ingredient and any processing or substitution. We distinguished a complete formula used as a clinical intervention from an isolated constituent used as a mechanistic probe and from a microbial metabolite proposed as an effector. For studies comparing different batches, manufacturers, extraction procedures, or dosage forms, chemical equivalence could not be assumed without comparative analytical data. When key information was absent, the study remained eligible but the limitation was recorded and the corresponding microbiota, bile-acid, or host-signalling findings were interpreted as product-specific and not automatically generalizable to the named formula as a whole.

Chemical standardisation defines the exposure being evaluated. Botanical identity, extraction, marker compounds, dose, batch comparability, contaminant testing, and administration and sampling timing should be reported before microbiota or bile-acid differences are interpreted. Detailed reporting criteria are consolidated in Section 3.1 and Table 4. Formula-level interpretation should distinguish the administered formula as a clinical preparation, an isolated constituent as a mechanistic probe, and a microbial metabolite as a candidate effector. A chemical fingerprint identifies the preparation but does not establish which constituent or microbial product drives the phenotype; conclusions should therefore remain at the level directly tested and should not be generalized across these units.

Microbiota dependence is a testable hypothesis rather than an assumption. We assign microbiota dependence only when depletion alters the phenotype, and we interpret FMT as evidence of transferability. Pathway-specific mediation requires measurement of a nominated microbial function and bile-acid intermediate in a relevant compartment. It also requires a pathway-directed perturbation, such as exposure-matched metabolite add-back, defined-strain or gene manipulation, isotope tracing, enzyme inhibition, or receptor perturbation, that changes or rescues the phenotype (Tawulie et al., 2023; Zhang H. Y. et al., 2021). Microbial and hepatic biotransformation further expands the set of bioactive entities under evaluation. A biotransformation-integrated network-pharmacology strategy, exemplified for Astragaloside IV and its metabolites, combined metabolite identification with experimental validation to distinguish parent-compound from transformed-product mechanisms (Hu et al., 2023).

### 3.2 A framework for mechanistic evaluation

Mechanistic evaluation follows one sequence: a chemically defined intervention and prespecified endpoint; function-resolved microbiome measurement; compartment-specific bile-acid profiling; receptor-proximal or cellular readouts; and pathway perturbation when mediation is proposed. This sequence is used to classify evidence in Section 2.3 and Figure 2.

There are published reports of possible activity with varying mechanistic support. In experimental metabolic settings, Jiang-Tang-San-Huang pill and modified Gegen Qinlian decoction have been associated with altered bile-acid metabolism and TGR5-associated signaling (Tawulie et al., 2023; Liu R. et al., 2024). Shengmai San, ZeXieYin, Qushi Huayu, and other formulae have been reported to alter microbiota and bile-acid measures while improving metabolic phenotypes (Wang Z. et al., 2024; Zhou S. et al., 2025). Banxia Xiexin decoction and Gegen Qinlian decoction have been linked to improved barrier or immune-metabolic pathways in colitis models (Luo et al., 2024; Wang X. et al., 2023). Whether these effects are mediated by a specific microbial bile-acid transformation remains unclear.

Probiotic fermentation of herbal materials is also under investigation to potentially modify the bioavailability of bioactive compounds and their relationship with the gut microbiota (Fan et al., 2025). Most literature available, however, is mechanistic and preclinical, and cannot be used to draw conclusions about clinical efficacy or a common bile-acid mechanism among the fermented products.

Representative TCM findings are summarized in Table 3; because most studies lack pathway interruption, they are interpreted as coordinated responses or hypotheses rather than proof of mediation.

Not reported in indexed record indicates that the item was not available in the primary article metadata or abstract assessed for this review; it is not evidence that the information is absent from all manufacturer or author records.

Intervention characterisation is an upstream requirement for causal interpretation, because a common formula name does not establish equivalent intestinal exposure. Three questions must be answered separately. Identity establishes what botanical material and final product were administered. Chemical comparability establishes whether independently released lots contain a sufficiently similar constituent profile. Exposure definition links the final product to its administered dose, schedule, and sampling window. Voucher-supported authentication, a final-product batch identifier, quantitative markers, an analytical fingerprint, predefined acceptance limits, and stability and contaminant testing are therefore not interchangeable reporting items. Together, they define whether the exposure can be reproduced and compared across studies (Heinrich et al., 2020; Xie et al., 2006).

To make this boundary operational, Table 4 assigns a reporting tier on the basis of information retrievable for this review. Tier I requires authenticated material and final product, lot-level traceability, quantitative marker data and an analytical fingerprint with prespecified acceptance limits, and evidence of between-batch comparability. Tier II indicates a partly characterised preparation, such as a partial chemical profile, without demonstrated lot-level chemical comparability. Tier III indicates that the report does not provide sufficient retrievable evidence for authentication, marker/fingerprint acceptance criteria, or batch comparability. These are reporting tiers, not estimates of biological activity or safety.

The tier determines the permissible level of inference. Tier I is necessary, but not sufficient, for a transferable formula-specific mechanism claim, which still requires the pathway evidence defined in Section 2.3. Tier II findings apply only to the described preparation. Tier III findings apply only to the tested product or batch. Neither Tier II nor Tier III evidence can establish an effect of the named formula, its constituent herbs, or TCM as a therapeutic class. A large downstream microbiota or bile-acid signal cannot remedy an incompletely defined exposure.

## 4 Metabolic disease applications: MASLD, obesity, and T2D

The disease-specific branches in Figure 1 emphasize that the consequences of microbial and bile-acid changes are context dependent across MASLD, obesity/T2D, and IBD.

Across MASLD, obesity/T2D, and IBD, microbial and bile-acid changes require disease-matched interpretation. In MASLD, weight loss or direct hepatocyte effects can improve liver endpoints independently of microbiota changes. In obesity/T2D, meal timing, metformin, diet, and caloric intake affect ileal FXR/TGR5 signalling, GLP-1, glucose disposal, and energy expenditure. In IBD, ileal absorption, surgery, transit, and biologic therapy can dominate luminal and mucosal signals. A TCM mechanism supported in one disease therefore requires disease-matched models, sampling compartments, and outcomes before extrapolation (Nie et al., 2024; Wang Q. et al., 2023; Schirmer et al., 2024; Vich Vila et al., 2024).

Across MASLD, obesity/T2D, and IBD, microbial BSH and hydroxysteroid-dehydrogenase activities can reshape the primary-to-secondary bile-acid balance and engage FXR/TGR5-linked metabolic or immune programs. Each setting should therefore measure microbial function, compartment-specific bile acids, barrier integrity, and receptor-proximal responses. The clinical meaning of a bile-acid change remains disease-specific: liver lipid accumulation and fibrogenesis in MASLD; postprandial GLP-1, energy balance, and thermogenesis in obesity/T2D; and ileal dysfunction, mucosal inflammation, and bile-acid reabsorption in IBD (Perino et al., 2021; Mohanty et al., 2024).

### 4.1 MASLD

MASLD involves diet, intestinal permeability, microbial metabolites, hepatic lipid metabolism, inflammation, and fibrosis. Altered secondary bile acids, impaired barrier function, and dysbiosis have been associated with disease severity, although bile-acid profiles differ among cohorts, disease stages, medication exposures, and sample types (Nie et al., 2024). Experimental studies provide more direct evidence that microbial secondary-bile-acid pathways can modify steatohepatitis in mice (Nie et al., 2024), whereas hyodeoxycholic acid (HDCA) has been associated with gut-liver protection in experimental MASLD (Kuang et al., 2023). These results suggest that targeting the gut-liver axis could be beneficial; however, a beneficial bile-acid profile is not universal (Lau et al., 2025; Long et al., 2024; Qu et al., 2023; Kuang et al., 2023; Schnabl et al., 2025).

Human evidence should guide TCM research in MASLD. Most mechanistic studies are conducted in diet-induced rodent models, while a randomized trial of Qushi Huayu has yielded clinical evidence of efficacy and safety (Liu Q. et al., 2024). Formula products like JiGuCao capsule, and their ingredients like baicalin and yellow-tea polysaccharides were linked to alterations in lipid metabolism, intestinal ecology, and bile-acid profiles (Qi et al., 2025; Huang Y. et al., 2024). It is important to determine if the microbial-bile-acid phenotype is associated with a response and if this proposed pathway is still linked to benefit when corrected for weight loss, energy intake, and co-treatments in future studies. They must also distinguish the hepatocyte-mediated effects from those mediated by microbiota (Cui et al., 2025; Hu et al., 2021; Huang L. et al., 2024; Wang H. et al., 2024; Zhang et al., 2024).

Clinical evidence for formulae should remain indication-specific. A recent meta-analysis with machine learning assessed Xiao-Chai-Hu Decoction across chronic liver disease settings, illustrating that a shared formula identity does not substitute for disease-specific efficacy and mechanistic evidence (Wang et al., 2025).

MASLD illustrates why a multilevel study design is necessary. Hepatic lipid handling, inflammation, fibrosis-related measures, intestinal permeability, and clinical safety may each improve without a microbial bile-acid mechanism. A convincing axis-based study should link a well-characterized intervention to a change in microbial activity and bile-acid composition in the relevant compartment, then test whether the proposed host pathway accounts for the hepatic phenotype. High-fat-diet models are useful for generating hypotheses but cannot substitute for data from patients with diverse diets, medication use, comorbidities, and disease stages. The aim is to identify microbial-metabolic phenotypes associated with benefit in a defined clinical setting, not to impose a single MASLD pathway (Wu et al., 2023; Smirnova et al., 2022; Wen et al., 2025; Long et al., 2025).

A disease stage, metabolic phenotype, medication history, and microbial-bile-acid phenotype would all be valuable in developing a stratification framework in MASLD. The role of secondary-bile-acid pathways and host responses could differ between steatosis, steatohepatitis and fibrotic disease. Thus, studies should be linked to clinically relevant liver outcomes and not act as a universal marker of benefit based on a single fecal signature of bile-acids or microbes. This method can discriminate between candidate effects on barrier function, hepatic lipid handling, inflammation or fibrosis from co-occurring changes due to weight loss or dietary adjustments (Nie et al., 2024; Fuchs et al., 2025; Zheng et al., 2024).

### 4.2 Obesity and T2D

In obesity and T2D, recurrent themes include TGR5-dependent GLP-1 release, FXR-TGR5 crosstalk, energy absorption, thermogenesis, and bile-acid composition (Wang Q. et al., 2023). Their relationships are not linear. Bariatric surgery, ketogenic diets, fiber interventions, hypoxia, and common antidiabetic drugs can change diet, transit, host metabolism, and microbiota at the same time. Bile-acid changes may therefore be mediators, consequences, or both. Measuring only fasting stool microbiota before and after a herbal intervention cannot distinguish these possibilities (Chen B. et al., 2023; Li et al., 2024a; Liu et al., 2023; Makki et al., 2023; Münzker et al., 2022).

Other gut-derived routes can modify metabolic outcomes independently of bile acids. In olanzapine-treated rats, lipid disturbance coincided with changes in short-chain fatty acid-related microbial features, caecal 5-HT and hypothalamic appetite signalling in a vagus-sensitive gut-brain setting (Zhu et al., 2022). TCM research in diabetic cardiomyopathy also emphasises direct host processes, including inflammation, oxidative stress, fibrosis, and AMPK, Nrf2 and NF-kB signalling (Li W. et al., 2024). These mechanisms define competing routes that should be measured alongside microbiota-bile-acid pathways.

The debate has gone beyond the canonical models of FXR and TGR5 in recent studies. Microbial control of bile-acid signaling can be modified by host metabolic state (Won et al., 2025). Microbial tryptophan-conjugated cholic acid was reduced in T2D and was beneficial for glucose tolerance in diabetic mice via MAS-related G protein-coupled receptor family member E (MRGPRE)-associated signaling (Lin et al., 2025). These observations lead to testable hypotheses of metabolites-receptors but do not provide evidence of the mechanism of action of a TCM intervention (Wu et al., 2021).

Explicit models can be tested in T2D with modified Gegen Qinlian decoction, Jiang-Tang-San-Huang pill, and edible TCM interventions (Liu R. et al., 2024; Tawulie et al., 2023). In the future, treatment trials should document diet and medication history, take serial stool and plasma samples, measure functional changes in BSH, *bai*, or other pertinent genes, and include measurements of glycemic outcomes and incretin/bile-acid profiles. When proposing a TGR5 mechanism, GLP-1 dynamics and receptor-proximal readouts should be defined *a priori*. These studies can determine whether TCM benefits a defined subgroup, for example as a microbiota-informed adjunct, or whether the same mechanism applies more broadly in T2D (Chen et al., 2024; Liu J. et al., 2025; Liu W. et al., 2025; Wang Y. et al., 2024; Chu et al., 2022).

There is currently preclinical evidence to support possible connections between the axis and skeletal-muscle metabolism. They should be explored as a separate hypothesis with muscle-relevant phenotypes and exposure measurements, rather than being added to liver or glucose claims without direct evidence (Mancin et al., 2023).

The newly identified amino-acid conjugates of bile acids expand the number of potential links between microbial metabolism and glucose homeostasis. They also show the shortcomings of taxonomy-only analyses: a named genus cannot determine the abundance, activity, or host significance of a certain metabolite. Targeted metabolomics and gene-resolved microbiome data should then be included together with the functional validation when the TCM intervention is associated with an emerging bile-acid pathway. This type of evidence is hypothesis-generating until the necessary links between the intervention, metabolite and receptor response, and outcome are established in the same experimental system (Lin et al., 2025; Won et al., 2025).

Energy intake, weight loss, physical activity, metformin and other treatments are major competing explanations for the changes in microbiota and bile acids for obesity and T2D. These exposures should be collected longitudinally during trials and the stool and plasma sampling matched to meals and dosing. However, if the release of GLP-1 is proposed to be TGR5 dependent, the study should prespecify bile-acid ligands, dynamic incretin measurements, and receptor-proximal readouts. The goal is to identify a subgroup where a specific intervention results in a change in a measurable pathway that leads to a better glycemic control, not the assumption that this would be common to all people with T2D (Wang Q. et al., 2023; Tawulie et al., 2023).

Although preclinical models are still useful for analyzing specific pathways, they must be interpreted with caution. Diet-induced models can be used to determine if a formula or a constituent alters any change in microbial function and bile-acid metabolism under controlled conditions. Human studies need to further consider heterogeneous diets, disease stage, polypharmacy, and safety. Plausible microbiota-dependent mechanisms should be separated from direct hepatobiliary or gut pharmacology with complementary perturbation experiments and by assessing for a baseline phenotype that predicts response in clinically relevant populations (Liu Q. et al., 2024; Qi et al., 2025).

Obesity is closely related to T2D but raises distinct interpretive problems. Changes in food intake, physical activity, metformin use, and other interventions can alter the microbiota and bile-acid pool while improving metabolism through independent pathways. TGR5-mediated GLP-1 release and FXR-TGR5 crosstalk are biologically plausible mechanisms, but taxonomy alone cannot demonstrate either process. Studies should also distinguish short-term post-treatment changes from durable microbial-metabolic phenotypes that may help stratify or monitor patients. When an intervention changes energy balance, a microbial signal may follow rather than mediate the metabolic response. Careful assessment of diet and co-medication is therefore required when interpreting TCM studies of obesity and T2D (Wang Q. et al., 2023; Tawulie et al., 2023; Li et al., 2023; Luo et al., 2025).

The gut microbiota-bile acid axis is also hypothesized to interact with skeletal-muscle metabolism, although this potential relationship is largely preclinical and not yet considered a clinical causal relationship (Mancin et al., 2023).

## 5 IBD: a priority setting for translational evaluation

IBD complicates evaluation of the microbiota-bile-acid axis because inflammation, ileal dysfunction, medication use, diet, and surgery can all alter both the microbiota and bile-acid pool. Multi-omics studies have linked microbial genes and metabolite programs with disease course and response to biologics. Clinical data also suggest that ileal disease, resection, and primary-bile-acid enrichment can modify inflammatory ecology (Vich Vila et al., 2024). In different models and anatomical sites, bile-acid receptor signaling has been linked to epithelial defense, immune-cell programs, and barrier repair (Han et al., 2023; Gallagher et al., 2021; Hernández-Rocha et al., 2021; Leibovitzh et al., 2024; Shi et al., 2023). As highlighted in Figure 1, inflammation, co-medications, intestinal transit, and disease stage can modify both the microbiota–bile acid pathway and its interpretation in IBD.

Specific bile-acid species and microbial programs can influence colitis outcomes in experimental models (Li T. et al., 2024). Dietary therapy, fecal microbiota transplantation (FMT) and biologic treatment also alter the microbial and metabolite profile and should be controlled (Lee et al., 2021). In experimental colitis, TCM formulae and components have been linked to barrier integrity, aryl hydrocarbon receptor (AhR)/interleukin-22 (IL-22) signaling, short-chain fatty acids (SCFAs), inflammasome pathways, and general modulation of microbial metabolism (Zhou et al., 2024). Most of the evidence is still preclinical, and the interventions and outcome measures are highly heterogeneous (Li et al., 2025; Pi et al., 2023; Wang M. et al., 2024; Zhou J. et al., 2025; Bretin et al., 2023).

Therefore, TCM may be evaluated as an adjunct to conventional induction or maintenance therapy in well-phenotyped IBD populations, rather than being presented as an alternative treatment. Trials should prespecify mucosal or symptom outcomes, safety surveillance, ileal versus colonic disease, previous surgery, bile-acid diarrhea, diet and concomitant biologic or small-molecule therapy.

Herbal colitis models are useful to test hypotheses related to barrier function, immune response and microbial metabolism, but the results cannot be extrapolated to clinical IBD. Banxia Xiexin decoction and Gegen Qinlian decoction were linked to the improvement of experimental colitis and regulation of microbiota-related pathways. Specific experiments are necessary to establish whether these effects are specific to a particular bile-acid transformation or to other microbial metabolites or directly to host pharmacology. Human studies should retain standard induction and maintenance therapy as the comparator and assess TCM as a phenotype-guided adjunct (Luo et al., 2024; Wang X. et al., 2023; Jing et al., 2025).

Dietary therapy, FMT, and biologics show that successful treatment can change microbial and metabolite profiles (Godny et al., 2025; Lee et al., 2021). These co-interventions should be recorded and included when evaluating TCM. A study protocol should specify the clinical outcome, treatment dose, adverse-event monitoring, and the microbial or bile-acid function proposed to mediate benefit. This is particularly important when symptoms improve before a durable mucosal or metabolic phenotype is established (Verburgt et al., 2023; Chen L. A. et al., 2023; Kedia et al., 2022).

Studies of this axis should first define the disease phenotype. Ileal involvement, colonic involvement, resection, inflammatory activity, diet, and biologic therapy can each alter bile-acid delivery and microbial ecology. Repeated sampling across clinical states is therefore preferable to a single post-treatment comparison. Stool is a poor surrogate when the proposed target is mucosal, ileal, hepatic, or systemic. The sampling matrix should match the biological claim, and the analysis should distinguish changes associated with disease control from those that contribute to it (Vich Vila et al., 2024; Battat et al., 2023; Lewis et al., 2024).

Evidence from extraintestinal inflammatory models also cautions against simple extrapolation. *Parabacteroides distasonis* has been reported to reduce the severity of experimental inflammatory arthritis, but this finding does not demonstrate an equivalent organism-level mechanism in IBD (Sun et al., 2023; Zhao et al., 2023; Lu et al., 2025).

A longitudinal pouchitis case study showed that repeated FMT was associated with transient engraftment of microbial functions such as butyrate metabolism, bile-acid metabolism, and tryptophan metabolism (Deng et al., 2023). This was a small case series and as such it is not necessarily a panacea for FMT in IBD.

IBD is a demanding test case because inflammation, ileal disease, surgery, diet, and therapy alter microbial ecology and bile-acid absorption. Trials should define phenotype, activity, surgery, and concomitant therapy, and recognize that ileal and colonic findings may not reflect the relevant mucosal compartment. TCM should be tested as a monitored adjunct to standard care, not as a replacement without comparable efficacy (Vich Vila et al., 2024; Lee et al., 2021).

Sampling design strongly affects inference in IBD studies. When the proposed mechanism concerns the ileum, mucosa, or systemic circulation, a post-intervention stool sample may reflect changes in transit, bleeding, inflammation, diet, or medication use. Repeated stool and plasma sampling can separate transient changes from persistent alterations in the bile-acid pool. Mucosal sampling may be informative when needed, but it does not replace luminal sampling. Clinical outcomes should be defined independently of the multi-omics analysis, and both safety and efficacy should be assessed. This design can show whether a microbiota-bile-acid signature is associated with disease control, treatment response, or resolution of inflammation (Schirmer et al., 2024).

## 6 Discussion

The minimum translational package is summarized in Table 5 and comprises authenticated, chemically fingerprinted material; exposure, diet and co-medication records; function-resolved microbiome and targeted bile-acid measurements; a nominated host pathway; disease-relevant outcomes; and prospective safety monitoring. The remaining discussion focuses on how this package should be implemented and interpreted.

**Table 5:** Minimum translational framework for TCM–microbiota–bile-acid studies, including dose/exposure, mechanism, evidence grading, safety and herb–drug interaction reporting.

Domain	Required report	Representative study/model	Dose and exposure requirement	Major microbiota/BA finding	Mechanistic test	Evidence level	Safety/interaction limitation
Intervention identity	Botanical authentication and voucher; final-product manufacturer and batch identifier; lot-specific quantitative markers; analytical fingerprint; predefined acceptance limits; stability/contaminant testing; and inter-batch comparability.	Qushi Huayu trial; JiGuCao and formula studies	State human g/day or animal g/kg, route, frequency and duration; provide conversion rationale	Only a Tier I intervention can support a transferable formula-specific mechanism claim. A common formula name alone cannot establish equivalent exposure.	Lot-matched replication across independently released batches.	Tier I required for transferable formula-level inference; Tier II/III are preparation- or batch-bounded.	Underspecified products prevent reproducibility
Model/population	Disease stage, diet, sex, age, medications and comparator	246-person RCT conducted under a historical NAFLD label	Dose must be linked to body weight, exposure and clinically relevant range	Baseline community and BA pool are major effect modifiers	Stratified or matched analysis	A–C	Rodent phenotypes and selected patients may not generalize
Microbiome and BA measurement	Shotgun/function assay plus targeted stool, plasma (and tissue when feasible) BA panel	Mechanistic comparators (Makki et al., 2023; Nie et al., 2024)	Prespecify sampling time relative to dosing and meals; quantify parent/product pairs	BSH, *bai* and hydroxysteroid dehydrogenase functions should be measured, not inferred from genus	Stable-isotope kinetic tracing or defined-strain colonization	B–C	Stool alone does not establish portal/tissue exposure
Causal validation	Microbiota depletion to test dependence; FMT to test transferability; exposure-matched metabolite add-back, defined strains, gene or enzyme perturbation, isotope tracing, or receptor perturbation to test pathway-specific mediation	TGR5/FXR pathway tests; microbial secondary-BA MASH models	Use exposure-matched add-back and pharmacodynamic confirmation	A nominated metabolite must track before host/outcome change	Depletion establishes dependence and FMT establishes transferability; add-back or pathway blockade is required to support mediation	B (if pathway-specific perturbation is complete) or C	Most TCM studies currently lack pathway-specific interruption
Clinical efficacy, safety and herb–drug interactions	Prespecified clinical endpoint, adverse-event coding, liver/renal tests, interaction adjudication and standard-care comparator.	Qushi Huayu RCT; future adjunctive trials	Report adherence, co-medications and cumulative dose	A biomarker shift is insufficient without clinical benefit beyond standard care	Longitudinal mediation and safety monitoring	A for efficacy; D for untested mediation	Small, short and unregistered studies limit translation; absence of a safety signal does not establish interaction safety across doses, batches, products or co-medications.

The comparison also defines a disease-specific translation strategy. A shared core package should quantify intervention chemistry, microbial function, targeted bile acids, FXR/TGR5 or immune readouts, and a prespecified clinical outcome. MASLD studies should additionally prioritize liver fat, inflammation and fibrosis with adjustment for weight change; obesity/T2D studies should prioritize standardized meal tests, GLP-1, insulin sensitivity and energy expenditure; and IBD studies should prioritize endoscopic or histologic inflammation, ileal status, surgery, transit and concomitant therapy. This framework prevents a generic “gut microbiota–bile acid” signature from being treated as a universal mechanism or therapeutic endpoint (Perino et al., 2021; Mohanty et al., 2024).

Across disease sections, unperturbed microbiota–bile-acid changes are interpreted as associations, while direct host pharmacology, diet, weight loss, co-medication and altered transit remain competing explanations (Agus et al., 2021; de Vos et al., 2022; Mohanty et al., 2024).

Pathway tests should be matched to the question: depletion assesses microbiota dependence but has host effects; FMT tests transferability but also carries donor- and host-mediated effects; defined strains or isotope tracing resolve microbial contribution; metabolite add-back or enzyme inhibition tests the candidate transformation; and receptor blockade tests host-node necessity. Depletion or FMT alone therefore cannot establish mediation by a specific microbial bile-acid transformation. Concordance across complementary tests is stronger than any single assay.

Precision includes safety. Bile-acid alterations may affect diarrhea, cholestasis, gallstone risk, drug disposition, mucosal inflammation, and cancer-related pathways (Režen et al., 2022). In patients receiving antidiabetic, immunomodulatory, or hepatology therapy, product variability and herb-drug interactions require an adjunctive strategy evaluated against standard therapy, not a blanket substitute.

Safety evidence should be considered separately from biological activity. TCM products may produce adverse effects through direct pharmacology, contamination or adulteration, batch-to-batch variability, and changes in bile-acid handling or intestinal drug absorption. Microbiota-mediated changes in drug metabolism and transport can modify exposure to co-administered agents, including antidiabetic drugs, statins, anticoagulants or antiplatelet agents, immunosuppressants, and biologics; inhibition or induction of CYP enzymes, P-glycoprotein, or other transporters should therefore be treated as a plausible interaction pathway rather than assumed to be clinically neutral (Chu et al., 2022; Foley et al., 2021). Potential pharmacodynamic interactions include additive hypoglycaemia, bleeding, hepatotoxicity, nephrotoxicity, or altered immunosuppression. These risks are particularly relevant when TCM is used as an adjunct, because an apparent improvement in a biomarker can coexist with a clinically important change in exposure to standard therapy.

Exploratory studies can be integrated into a staged translational pathway. Chemical comparability, exposure and reproducible microbial-metabolic phenotype should be determined in early studies. The predictions of pathways should then be tested using targeted metabolomics and functional perturbation in mechanistic studies. The phenotype should be tested in larger trials to determine if it is clinically useful in addition to standard therapy, and if it is effective across batches, populations and disease stages. Negative results still have information value, if the intervention, pathway and endpoints were predetermined.

For future adjunctive trials, safety should be prospectively defined and monitored rather than reported only as a general statement of tolerability. Investigators should document all prescription drugs, over-the-counter products, supplements, alcohol use, and herbal products; record batch and dose exposure; and prespecify adverse-event coding, discontinuation criteria, and interaction adjudication. Baseline and serial alanine aminotransferase, aspartate aminotransferase, alkaline phosphatase, bilirubin, creatinine/eGFR, glucose, and—when clinically indicated—coagulation and drug-concentration measurements should be aligned with the proposed mechanism. Liver injury, cholestasis, diarrhea, bleeding, hypoglycaemia, renal injury, and disease flares should be actively queried. A conventional-treatment control and an adjunctive-treatment arm are needed to determine whether TCM adds benefit without increasing toxicity; short, underpowered studies cannot establish interaction safety (Farooqui and Elhence, 2022; Režen et al., 2022).

Safety should be evaluated independently of biologic activity. Bile-acid perturbation and herb-drug interactions require prespecified laboratory monitoring, interaction adjudication, and adverse-event reporting; a biomarker change or no signal in one trial does not establish safety across products or populations (Farooqui and Elhence, 2022; Liu Q. et al., 2024). Studies should prespecify falsifiable predictions linking intervention markers, microbial activity, bile-acid species or ratios, host signals, and clinical endpoints; failed predictions refine the mechanism. Replication across batches, populations, and disease stages is required before clinical selection or monitoring. Qushi Huayu reported efficacy and no new serious safety signal, but not interactions with conventional therapies. Broad adjunctive use requires pharmacovigilance and transparent reporting of withdrawals and suspected herb-drug interactions.

Reproducibility begins with chemically equivalent lots, function-resolved microbiome analyses, and bile-acid assays that specify species and compartment. Samples should be linked to dosing, feeding, and disease activity; analytical pipelines must report extraction, sequencing, mass spectrometry, reference materials, and handling of batch effects, missing values, compositional data, and multiple testing. Multi-omics should follow the prespecified pathway, with independent cohorts confirming between-site reproducibility and value beyond clinical variables, diet, medication use, and disease activity. Early trials can test biological activity, whereas later trials test prespecified clinical effects against standard care. Stratified randomization, longitudinal exposure assessment, and transparent adherence and adverse-event reporting remain essential.

## 7 Conclusions and future directions

The distinctive contribution of this review is to recast TCM research on the gut microbiota-bile acid axis as an evidence-calibrated translational problem rather than a catalogue of dysbiosis signatures. We integrate four elements that are often considered separately: chemical and dose standardization of complex TCM interventions; function- and strain-resolved microbial biology; compartment-specific bile-acid exposure and receptor-proximal host responses; and pathway-directed validation linked to disease-relevant outcomes. This synthesis identifies where current evidence is associative, where it is mechanistically supported, and where it meets the predefined criteria for pathway-supported inference. It also makes explicit that the same microbial or bile-acid signal can have different meanings in MASLD, obesity/T2D, and IBD.

Clinical evidence remains sparse. Only one eligible human clinical trial, the Qushi Huayu study in adults enrolled under the historical NAFLD designation, reported both clinical outcomes and microbiota-related outcomes. Evidence for obesity/T2D and IBD was predominantly preclinical or observational, and no eligible human comparative study combined a defined TCM exposure with microbiota–bile-acid measurements. The framework therefore provides a structure for translational evaluation rather than clinical validation of TCM–microbiota–bile-acid mechanisms.

The gut microbiota–bile acid axis provides a testable framework for evaluating TCM in metabolic and inflammatory diseases. Rather than broadly claiming microbiota regulation, it asks whether a chemically defined exposure alters a specific microbial function or bile-acid trajectory, engages a host response and improves a disease-relevant outcome. Strong evidence integrates longitudinal measurements, functional microbiology, compartment-specific bile-acid profiling and pathway perturbation. Depletion tests microbiota dependence and FMT tests transferability, but both interventions alter host physiology and immunity, so neither intervention alone can identify a specific mediator. Mediation requires measurement of the candidate transformation plus complementary validation, such as metabolite add-back, defined-strain or gene manipulation, isotope tracing, enzyme inhibition or receptor perturbation. Convergent tests provide stronger support for mediation.

The same criterion should be used for reading the respective signals in MASLD, obesity/T2D, and IBD, and the biological relevance of the signal must be understood with respect to the disease stage, anatomical compartment, co-medications, concurrent treatment, and diet. The information on a change in relative abundance or on a single bile acid may be useful for hypothesis generation, but it is not adequate to determine a producer, establish enterohepatic flux, or infer a therapeutic mechanism. In particular, for TCM, the formulae might act directly on the host tissues and the product composition, extraction, dose and batch consistency all influence the intestinal exposure. The strength of the conclusion should therefore reflect the intervention, compartment and causal test used.

Chemical incompleteness is therefore not a peripheral reporting limitation. If botanical identity, final-product composition, lot-specific marker values, or batch acceptance criteria are unavailable, equivalent intestinal exposure cannot be confirmed. Under those conditions, an observed microbial or bile-acid change cannot be attributed reliably to the named formula, pooled across nominally similar products, or generalized to clinical use. The interpretation must remain bounded to the tested preparation or batch until Tier I characterisation and pathway validation are available.

Future work should prioritize validated interventions, longitudinal human studies and clinically meaningful endpoints, with strain- and gene-resolved microbiome data, targeted stool/plasma bile-acid panels, standard-care comparators and interaction-aware safety monitoring. TCM is therefore best positioned as a phenotype-guided adjunct until reproducible benefit and mediation are demonstrated.

The translational value is therefore practical: the proposed evidence hierarchy and minimum reporting package provide investigators with falsifiable criteria for designing experiments, interpreting null results, selecting responsive phenotypes, and deciding whether a TCM product is ready for adjunctive clinical testing. By requiring benefit beyond standard care together with reproducible exposure, predefined pathway evidence, and interaction-aware safety monitoring, the framework limits overinterpretation while preserving clinically useful hypotheses. The review thereby contributes a route from mechanistic discovery to responsible, phenotype-guided TCM translation.
